# Molecular identification of *Toxoplasma gondii* in domesticated and broiler chickens (*Gallus domesticus*) that possibly augment the pool of human toxoplasmosis

**DOI:** 10.1371/journal.pone.0232026

**Published:** 2020-04-22

**Authors:** Muhammad Bar Khan, Sanaullah Khan, Khair Rafiq, Shahid Niaz Khan, Sobia Attaullah, Ijaz Ali

**Affiliations:** 1 Department of Zoology University of Peshawar Khyber Pakhtunkhwa Pakistan, Peshawar, Pakistan; 2 Department of Zoology Kohat University of Science & Technology Kohat Pakistan, Kohat, Pakistan; 3 Department of Zoology Islamia College Peshawar Khyber Pakhtunkhwa Pakistan, Peshawar, Pakistan; 4 Department of Biosciences, COMSATS University Islamabad Pakistan, Islamabad, Pakistan; Qatar University, QATAR

## Abstract

*Toxoplasma gondii* (*T*. *gondii*) is a protozoan parasite that infects all warm-blooded animals including domesticated birds and humans. Birds normally get infected by ground feeding and human beings contract the disease by consumption of undercooked chicken meat. This study aimed to analyze seroprevalence and DNA of *T*. *gondii* in chickens (domesticated and broiler) and to assess possible transfer to humans by review of available literature from Pakistan. Blood from and tissues from domesticated and broilers chickens were analyzed for *Toxo-*IgM/IgG and *Toxoplasma* DNA through ELISA and PCR respectively. Furthermore, research articles published during 1990–2019 on the prevalence of *T*. *gondii* in humans from Pakistan, were analyzed to assess the possible infection burden in the area in connection to transmission from chickens. The overall prevalence of IgM and IgG for *T*. *gondii* was 17.83% and 8.8% respectively in the study areas. Significant seroprevalence was found in domesticated chickens than broilers. In domesticated chickens, the prevalence was high in age ≥ 2 years. *Toxoplasma* DNA was detected in tissues with an overall prevalence of 10.84%. Higher prevalence was observed in liver (10.50%) than heart (9.5%) and muscles (7.11%). Only 4.78% broiler and 2.38% domesticated chickens were positive for both IgM and DNA, 1.2% domesticated and 1.30% broilers were positive for IgG and DNA, while 2.98% domesticated and 2.17% broilers were positive for IgM, IgG, and DNA. Available literature showed that 25.8% of human beings were infected with *T*. *gondii* in Pakistan. The prevalence was 20.64% in male and 26.82%in the female. The rate of infections increases with age and high (37.36%) was found in humans of age range 40 to 60 years. A high prevalence of *T*. *gondii* is found in both domesticated and broiler chickens in the study area. Moreover, the literature survey indicates that a high seroprevalence of *T*. *gondii* is present in human beings of Pakistan. It is concluded that the high prevalence of *T*. *gondii* in humans may be associated with the parasite transmission through infected chicken’s meat in Pakistan.

## Introduction

Toxoplasmosis is a zoonotic disease caused by a unicellular, protozoan parasite *Toxoplasma gondii* (*T*. *gondii*). It is a cyst-forming coccidian parasite and exists in three infectious morphological forms; i) an aggressive and quickly dividing tachyzoite stage, ii) slowly dividing semi-dormant bradyzoite stage within tissue cysts [1,2] and iii) an environmentally resistant sporozoite stage within oocysts. Sporulated oocysts survive for a long time under moderate environmental conditions and can infect all warm-blooded animals including birds and humans [3–5]. About one-third of the human population is infected by *T*. *gondii*, causing various complications like microcephaly, hydrocephaly, chorido-retinitis, mental health disorders [1] severe congenital pathologies and spontaneous abortion, or stillbirth [6]. Even in developed countries like the USA, annually 400–4000 infants are born with congenital toxoplasmosis and reports showed complications like schizophrenia, depression, and obsessive-compulsive disorder which are linked to *T*. *gondii* [7].

Various domesticated and farm animals and birds harbor infection and maybe a potent source of transmission to humans. *T*. *gondii* prevalence in different birds has been reported from various countries [8]. It is possibly transmitted to birds through food taken from the ground contaminated with oocysts. Free-range (domesticated) chickens may get infected with *T*. *gondii* during feed on the contaminated ground/soil with cat feces and excreta of domesticated animals. Broiler chickens may also become infected due to poor hygienic conditions in poultry farms [9]. Humans become infected by use of contaminated water and consumption of oocysts contaminated food. Direct consumption of undercooked chicken meat or meat products may be a possible way of *T*.*gondii* transmission to humans [10].

Humans consume meat as the main source of protein and global annual per capita meat consumption is expected to reach 35.3 kg by 2025. In Pakistan 1.02 billion broiler chickens are produced annually and it ranks the 11^th^ largest poultry producer in the world. In Pakistan poultry is the cheapest and favorite source of meat which contributes about 28% of the total meat production [11,12], so to meet the increasing demand of protein to the population, the pressure on the livestock industry is also increasing. It is estimated that per capita meat consumption in Pakistan increased from 11.7 kg in 2000 to 32 kg in 2016, almost double what was predicted that it will increase to 47 kg by 2020 [11]. It is reported that about 50% of all human *T*. *gondii* infections are foodborne [13], and 30–63% of infections are associated with the consumption of meat [1]. Some recent reports from Pakistan showed the prevalence of *T*. *gondii* in human population range from 12–28% [14–16]. This study was, therefore, designed to analyze *T*. *gondii* in blood and tissues of domesticated and broiler chickens and to associate it with the prevalence of infection in human beings in the study area.

## Materials and methods

### Study area

Two districts (Upper Dir and Peshawar) of Khyber Pakhtunkhwa province, Pakistan, were selected for this study. The district Upper Dir lies between 35°-04 to 35°-46 North latitudes and 71°-32 to 72°-22 East longitudes. The mean maximum and minimum temperature during June is about 33°C and 16°C respectively. The summer season of district Upper Dir is moderate and warm with June and July as the hottest months. The winter season is very cold and severe. The temperature rapidly falls from November till the end of March. During December-to February temperature falls below freezing point. The district Peshawar lies between 33°-44 and 34°-15 North latitudes and 71°-22 and 71°-42 East longitudes. Winter months in Peshawar start from mid-November and lasts till the end of March. The summer months are from May to September. The mean range of temperature in summer is 25–40°C [17]. The above mentioned two districts were selected because of different climatic conditions, cat densities and meat feeding habits of people. Blood and tissues were collected and analyzed at Molecular Biology and Virology Laboratory Department of the Zoology University of Peshawar, Khyber Pakhtunkhwa, Pakistan.

### Ethical approval

This study was approved by the Ethical Committee of the Center of Biotechnology and Microbiology, and Advanced Studies and Research Board University of Peshawar Khyber Pakhtunkhwa Pakistan. Prior verbal consent was taken from each farmer before samples and data collection.

### Sample and data collection

Data regarding food, feeding and living habit, cat presence in the vicinity, etc. was collected from farmers through a predesigned questionnaire. About 2mL blood was collected in a kindly manner to minimize pain from the jugular vein through a sterile syringe from 398 chickens (168 domesticated free-range and 230 broilers). The serum was separated and stored at -20°C until use.

Tissue samples were collected from 295 chickens (65 domesticated and 230 broilers) from which serum was already collected, in sterile plastic bags and transferred in cold box to the laboratory for further analysis. Domesticated chickens were purchased and slaughtered with a sharp knife without stunning in a humanely way and tissues (liver, heart, and pectoral muscle) were isolated, while tissues of broiler chickens were collected from poultry meat shops where chickens were slaughtered (with a sharp knife without stunning) and sold for commercial purposes.

### Serological examination

ELISA was performed using Bio-ELISA Toxo-IgM and IgG kits (Biokit, S.S. Barcelona Spain) according to the supplier’s instructions.

### DNA Extraction, amplification, and detection

The tissue specimen (~200mg) was macerated with a mortar and pestle in liquid nitrogen and then DNA was isolated using a DNA extraction kit (DNAzol® Reagent Thermo Fisher USA) according to the manufacture’s protocol. A highly conserved 35- fold repeats of the B1 gene of *T*. *gondii* DNA was amplified through nested PCR, using gene-specific primers (Toxo B22 (F): 5′AACGGGCGAGTAGCACCTGAGGAG′3) and (Toxo B23 (R): 5′TGGGTCTACGTCGATGGCATGACAAC′3) according to the method described by Sadek et al. [18]. Briefly, a PCR reaction of 20μL consisted of 10X *Taq* buffer, 1.2mM of MgCl_2_, 2μM of each primer, 0.2μM of each dNTP, 1U of *Taq* DNA polymerase (Thermo Fisher USA) and 5μL extracted DNA. Samples were initially incubated at 95°C for 5min, then 30 cycles of 95°C for the 30s, 60°C for 30s, 72°C for 60s and finally at 72°C for 10 minutes. The amplified DNA fragment of115bp (S1 Fig) was electrophoresed in 2% agarose gel, and visualized under UV light transillumination.

### Literature search

To find out the prevalence of *T*. *gondii* in humans, research articles were searched by using terms like *Toxoplasma gondii*, *T*. *gondii*, Human Toxoplasmosis, Seroprevalence, ELISA, PCR, and Pakistan. The articles were searched by using search engines like Google Scholar, Pubmed and Science Direct, which were published on the prevalence of *T*. *gondii* in humans, from the year 1990 to 2019. Forty-two research articles, relevant to the research work, were analyzed for *T*. *gondii* prevalence in humans in different provinces [Khyber Pakhtunkhwa (N = 20), Punjab (N = 18), Sindh (N = 3) and Baluchistan (N = 1)] of Pakistan. Of the total, only 37 could fulfill the required criteria and were included in this study.

### Statistical analysis

Statistical analysis of frequencies was calculated using the chi-square test (χ^**2**^) through SPSS version 20 for windows and the differences were considered statistically significant at <0.05.

## Results

### Seroprevalence of *T*. *gondii* in chickens

Of the total chickens, 17.83% (71) were IgM and 8.8% (35) were IgG positive while 5.77% (23) were both IgM and IgG positive. In domesticated chickens, 26.2% (44) were IgM and 14.88% (25) were IgG positive, while out of broiler chickens, 11.73% (27) were IgM and 4.34% (10) were IgG positive. Significantly (*P* = 0.02) high seroprevalence was found in domesticated chickens as compared to broiler chickens. In domesticated chickens, significantly high prevalence (*P* = 0.01) was found in female chickens (28.7% IgM, 16.7% IgG) as compared to males (21.7% Ig, 11.7% IgG). In broilers chickens, no significant difference was found in females (11.12% IgM, 5.18% IgG) and male (12.63% IgM, 3.5% IgG). In the current study, the prevalence rate increased with age significantly (*P* = 0.03) and high prevalence was found in domesticated chickens of age more than two years and in broilers of age more than 90–120 days. In domesticated chickens, high seroprevalence [IgM (28.7%), IgG (16.67%)] was observed in district Dir as compared to [IgM (21.67%), IgG (11.67%)], but the case was opposite in broiler chickens, where seroprevalence [IgM (8%), IgG (3%)] was low in Dir than Peshawar [IgM (14.61%), IgG (5.38%)] (Table 1).

**Table 1:** Seroprevalence of *T*. *gondii* in chicken (N = 398).

Variable (N)		Type	Total N	IgM +ve N (%)	IgG +ve N (%)	*P* Value
Overall		Domesticated	168	44 (26.20)	25 (14.88)	0.02
		Broilers	230	27 (11.73)	10 (4.34)	
		**Total**	**398**	**71 (17.83)**	**35 (8.80)**	
Gender	Male (155)	Domesticated	60	13 (21.70)	7 (11.70)	0.01
		Broilers	95	12 (12.63)	3 (3.15)	
	Female (243)	Domesticated	108	31 (28.70)	18 (16.70)	
		Broilers	135	15 (11.12)	7 (5.18)	
Age	Domesticated	< 1Year	32	6 (18.75)	3 (9.38)	0.03
		≥1Y &<1.5 Y	20	4 (20.00)	2 (10.00)	
		≥1.5 Y&<2 Y	52	12 (23.08)	8 (15.38)	
		≥ 2 Years	64	22 (34.37)	12 (18.75)	
	Broilers	30 to 60days	139	5 (03.60)	-	
		60 to 90days	59	12 (20.33)	4 (6.80)	
		90 to 120days	32	10 (31.25)	6 (18.75)	
Area	Dir Upper	Domesticated	108	31 (28.70)	18 (16.67)	-
		Broilers	100	8 (8.00)	3 (3.00)	
	Peshawar	Domesticated	60	13 (21.67)	7 (11.67)	
		Broilers	130	19 (14.61)	7 (5.38)	

### Evaluation of chickens’ tissues for *T*. *gondii* DNA

Of the total tissue samples (65 domesticated and 230 broilers) 10.84% (32) were found positive for *Toxo*-DNA. Non-significant (*P* = 0.25) high prevalence (10.50%) of *Toxo-*DNA was observed in the liver as compared to heart (9.5%) and pectoral muscles (7.11%). Comparatively, the high prevalence was found in tissues of domesticated chickens as compared to broiler chickens. *Toxo*-DNA was detected in 9.32% (11/118) male chickens and 11.87% (21/177) female chickens. High prevalence was found in females of both domesticated and broiler chickens (Table 2).

**Table 2:** Distribution of *T*. *gondii* DNA in various tissues and gender of chicken studied (N = 295).

Categories		Domesticated chickens		Broilers Chickens		Total PCR +ve N (%)	*P* Value
		N	PCR +ve N (%)	N	PCR +ve N (%)		
Overall		65	13(20.00)	230	19(8.26)	32(10.84)	0.02
Tissue	Liver	65	13(20.00)	230	18(7.82)	31(10.50)	0.12
	Heart	65	12(18.46)	230	16(6.95)	28(09.50)	
	Muscles	65	08(12.30)	230	13(5.65)	21(07.11)	
Gender	Male (N = 118)	23	04 (17.4)	95	07 (7.4)	11(09.32)	-
	Female (N = 177)	42	09 (21.4)	135	12 (8.9)	21(11.87)	

### Comparison of *Toxo-*antibodies and *Toxo*-DNA in chickens

Based on the overall results obtained, eight categories to compare antibodies and DNA absence/presence in domesticated and broiler chickens. Of the total *Toxo-*DNA detected in tissues 20% were in domesticated and 8.26% in broiler chickens. About 2.98% domesticated and 2.17% broiler chickens were positive for IgM/IgG and PCR. Complete results of all the eight categories are given in Table 3.

**Table 3:** Comparative analysis of *Toxo-*IgM, IgG and *Toxo*-DNA prevalence in chickens.

Chicken		Category*							
		I	II	III	IV	V	VI	VII	VIII
**Domesticated**	**N**	7	24	2	11	2	4	5	113
	**%**	4.16	14.28	1.20	6.54	1.20	2.38	2.98	67.26
**Broilers**	**N**	-	9	-	2	3	11	5	200
	**%**	-	3.91	-	0.87	1.30	4.78	2.17	86.95

* cat-I = IgG+ve, IgM-ve, PCR-ve, cat-II = IgG- ve, IgM+ ve, PCR- ve, cat-III = IgG- ve, IgM- ve, PCR+ ve, cat-IV = IgG+ ve, IgM+ ve, PCR-v, cat-V = IgG+ ve, IgM-v, PCR+ ve, cat-VI = IgG- ve, IgM+ ve, PCR+ ve, cat-VII = IgG+ ve, IgM+ ve, PCR+ ve, cat-VIII = IgG- ve, IgM- ve, PCR- ve

### Risk factors associated prevalence of *T*. *gondii* in chickens

The highest prevalence (40%) was found in those domesticated chickens living in the vicinity of cats. The overall *T*. *gondii* prevalence was found in domesticated chickens that were ground feeders and free-living. Low prevalence was found in the broiler chickens, as they were normally caged with no direct contact with cats, and fed in special pots (Table 4).

**Table 4:** Risk factors associated prevalence of *Toxoplasma* in chickens.

Risk Factors		Domesticated		Broilers	
		Total (N)	Positive N (%)	Total (N)	Positive N (%)
Cats nearby	Yes	135	54 (40)	-	-
	No	33	6 (18.18)	230	23 (10.00)
Feeding Habit	Ground feeding	168	60 (35.71)	-	-
	Feeding in pots	-	-	230	23 (10.00)
Living Habits	Free Living	168	60 (35.71)	-	-
	Caged	-	-	230	23 (10.00)

### *T*. *gondii* in humans: A literature review

Prevalence of *T*. *gondii* in humans was 25.8% (3636/14098), analyzed from available research articles (N = 41) from Pakistan. Overall, high prevalence (26.82%, 3150/11744) was observed in females as compared to males (20.64%, 486/2354).

Of the total, 21 research articles presented data of Khyber Pakhtunkhwa province, in which 16 articles showed data according to age. Male to female infection ratio was 1:8.1 and high prevalence was (32% was found in the age group 41–60 years. Overall, 19.8% (231/1168) males and 25.72% (1191/4630) females were positive for *T*. *gondii* reported from Punjab province. Of the total 16 articles from Punjab, only 14 mentioned age-relevant prevalence. In Sindh province, 40.22% (35/87) males and 47% (171/364) females were positive, while in Balochistan only five females were reported positive. Articles presented data of Sindh and Baluchistan did not show age-wise prevalence (Table 5).

**Table 5:** Prevalence of humans’ toxoplasmosis in different provinces of Pakistan.

Categories (References)		Gender			Age groups (years)				
		Male	Female	Total	01–20	21–40	41–60	61–80	>80
Khyber Pakhtunkhwa [16, 19–38]	N	1099	6745	7844	1118	4557	597	114	-
	+ve	220	1783	2003	254	1356	191	15	-
	%	20.01	26.43	25.53	22.7	29.75	32	13.15	-
Punjab [14,15,36,38–50]	N	1168	4630	5798	1186	2632	1030	150	12
	+ve	231	1191	1422	275	652	417	48	4
	%	19.8	25.72	24.52	23.18	24.77	40.48	32	33.33
Sindh [38,51,52]	N	87	364	451	-	-	-	-	-
	+ve	35	171	206	-	-	-	-	-
	%	40.22	47	45.67	-	-	-	-	-
Balochistan [38]	N	-	5	5	-	-	-	-	-
	+ve	-	5	5	-	-	-	-	-
	%	-	100	100	-	-	-	-	-
Overall Prevalence	N	2354	11744	14098	2304	7189	1627	264	12
	+ve	486	3150	3636	529	2008	608	63	4
	%	20.64	26.82	25.8	23	27.93	37.36	23.86	33.33

## Discussion

The chicken’s tissues can harbor *T*. *gondii*, promoting zoonotic transmission by consuming raw or undercooked meat [1]. In Pakistan, grilled chicken is a food of choice and traditionally free-range chicken is considered the healthiest and wholesome meat especially recommended for pregnant women and inevitably leads to public health risks. Free-range chickens can get *T*. *gondii* from contaminated soil during feed and are potential risks to humans [8]. About one third of the global population are positive for *T*. *gondii* antibodies [53], indicating worldwide spread [54]. Meat-borne infection previously reported from some countries was approximately 30% to 63% and was linked to eating habits, environment, housing as well as climatic conditions [54,55]. It is important to uncover the parasite pervasiveness in understanding the transmission patterns between man and animals [56]. The present study analyzed chickens and their tissues as a possible risk of *T*. *gondii* transmission to humans. It is observed that domesticated chickens harbored more *T*. *gondii* infection than broiler chickens. The high prevalence, in domesticated chickens, may be due to their frequent contact with cats and ground feeding. The low prevalence in broiler chickens may be attributed to their confinement, fast-breeding and few chances of contact with reservoir animals like cats [6,8]. However, it is also alarming that 10% of broiler chickens were positive for *T*. *gondii* and indicates flaws in existing poultry management and hygiene which needs improvement in the study area. It is also investigated during meta-analysis of bird toxoplasmosis in Iran that industrially raised chickens were significantly less infected as compared to free-range birds as their food and water were free of parasites and rare contact with cats or other animals [53].

Serum IgG and IgM immune assays are used to differentiate chronic and acute infection and population surveillance of *T*. *gondii* [7]. In the current study, 17.83% of chickens were found positive for IgM, 8.8% for IgG and 5.77% were both IgM and IgG positive. In the current study gender-wise prevalence in domesticated chickens were significantly (*χ*^2^ = 0.01, P<0.05) higher in females (28.7% IgM, 16.7% IgG) than males (21.7% IgM, 11.7% IgG). These findings are similar to those previously reported from different regions of the country [9,55]. Studies from different parts of the world have demonstrated parallel findings in other animals such as mice [57], dogs [58], goats [56,59], cats [53] and sheep [59]. The investigations demonstrated that gender influenced the incidence, severity, and course of *T*. *gondii* infection especially in female mice with severe brain inflammation [60], and severe combined immunodeficiency [61], while enhanced innate immune responses were reported in males [62]. It is also showed that the gender-based difference in responses to *T*. *gondii* is due to hormonal profiles [51,54,57,63], and reduced females immunity due to nutrition [63,64], age [54, 63], reproductive and certain environmental factors [7, 63,64].

In the current study, the prevalence of *T*. *gondii* was found to increase significantly (*χ*^2^ = 0.03, P<0.05) with age and high prevalence (28%) was found in domesticated chickens more than two years and 15.62% in broiler chickens old 90 days to 120 days. An increase in prevalence with age was also described in the previous studies, where older birds were at risk of exposure and more opportunities to come in contact with the contaminated environment than the younger ones [1,56,63,65,66]. High prevalence in older animals may also be due to their weak immune systems than younger animals [57].

Geography and environment also play an important role and considered major risk factors in the distribution of *T*. *gondii* infection [67]. In this study, high prevalence (28.7% IgM, 16.67% IgG) was observed in domesticated chickens from district Upper Dir when compared to Peshawar district (21.67% IgM, 11.67% IgG). District Upper Dir has high annual rainfall, and humidity which are considered favorable for the survival of *T*. *gondii* oocysts [1,65]. On the other hand, high prevalence of *T*. *gondii* in broiler chickens (14.61% IgM, 5.38%IgG) was reported from Peshawar as compared to district Upper Dir (8% IgM, 3% IgG), and it may be due to their excessive use, hygienic condition and dry climate which influence on the sporulation of oocysts in the environment [1]. The current study findings of the two different districts are in line with other studies who attributed prevalence in different geographical areas may be due to different social and cultural habits, sample size, and age, chicken housing, hygienic standards, drinking water type, cat densities, type of confinement, ecological and climatic conditions, and annual rainfall [1,63,68,69].

In this study, an overall 10.84%, *Toxo*-DNA was detected by PCR in the tissues of chickens. *Toxo-*DNA prevalence (10.84%.) of the current study is higher than reported from neighboring countries like India (6.06%) [70] and Iran (8%) [71], but lower than reported from Kenya (24–79%) [63, 72], and Iraq (26.9%) [73]. The change in prevalence might be due to the differences in geography, climate, the number and age of chickens examined, and sanitation conditions [68]. The highest prevalence (20%) was detected in the meat of domesticated chickens due to their free-living habit and possibly higher chance of contact with cat feces [1,9,63,71]. A high prevalence (10.50%) of *Toxo-*DNA was found in the liver tissues followed by heart and muscle (9.5% and 7.11%, respectively). The prevalence of the pathogen in chicken’s muscles of the current study was almost similar to a study from Canada [74] but lower from that of Brazil [75]. In the current study, *T*. *gondii* prevalence was more in older chickens and in those who were in contact with cats, free-living, and ground feeders, supported the investigations that cats, the only host can shed oocysts (resistant stage) in the feces hence increases the chances of *T*. *gondii* infection [4,5].

The prevalence of *T*. *gondii* in humans in Pakistan, assessed from literature was 25.8%. A high prevalence of 26.82% was found in human females as compared to 20.64% in males. Prevalence was found to increase with age and a high prevalence of 37.36% was found in humans of age range 40 to 60 years.

Pakistan is ranked in the largest poultry producer countries and also chicken is the easiest source of meat, contributes about 28% in the total meat production [11,12]. The increasing trends of chicken consumption [11] seem an increase in the chances of *T*. *gondii* transmission to the consumers. Previously, it is reported about 50% of all human *T*. *gondii* infections are linked to various food [13], meat contributed about 30–63% of infections [1]. It is worthwhile that the rise in chicken consumption in Pakistan [11,12] and the current study findings of high prevalence in chickens and humans may be associated with the possible transmission of *T*. *gondii* to humans.

## Conclusion and recommendations

High seroprevalence and *Toxo-*DNA were observed both in domesticated and broiler chickens. Furthermore, the literature review showed that toxoplasmosis is prevalent in humans in Pakistan. It is also concluded that the high prevalence of *T*. *gondii* in humans may be associated with the parasite transmission through infected chicken’s meat. The study suggests that chicken should be kept in a clean area and limit their contact with cats to reduce the chances of parasite transmission. Humans should avoid eating raw and undercooked chicken meat.

## Supporting information

S1 Fig Amplified DNA (115bp) of *T*. *gondi*: Left to right Lane 1& 3–6 positive, Lane 2 negative, Lane 7 negative control (distilled water), Lane 8 ladder marker (100bp). (PDF)
