# Supplementary figures and images for: Molecular identification of Toxoplasma gondii in domesticated and broiler chickens (Gallus domesticus) that possibly augment the pool of human toxoplasmosis
Source: PLoS One. 2020 Apr 22;15(4):e0232026. doi: 10.1371/journal.pone.0232026 (PMC7176139; doi:10.1371/journal.pone.0232026)

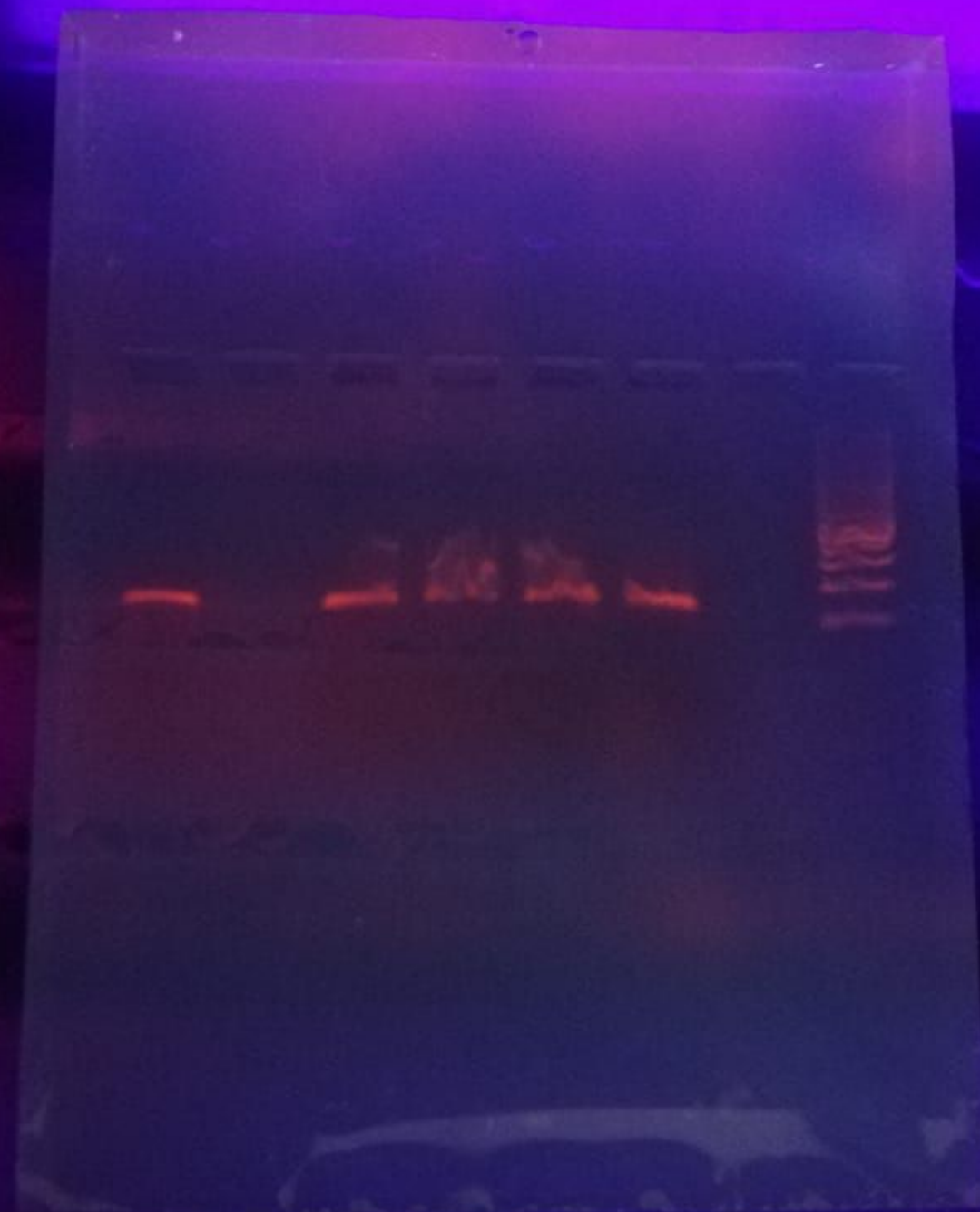

Supplement: S1 Fig — (PDF) [file pone.0232026.s001.pdf]
